# Supplementary material for: Are carbon emissions trading and green financial instruments synergistic? -Comprehensive quantitative research based on content analysis
Source: PLoS One. 2024 Mar 7;19(3):e0298601. doi: 10.1371/journal.pone.0298601 (PMC10919601; doi:10.1371/journal.pone.0298601)
Supplement: S1 Data — (ZIP) [file pone.0298601.s001.zip › Data/地级市Z_F工作报告.zip!/╔┬╬≈/▒a╝a╩╨/└┤╫╘╣1⁄2╓┌║┼í╛┬φ┐╦╩2╛▌═°í┐_2019.docx]

各位代表：

现在，我代表市人民政府向大会作工作报告，请予审议，并请市政协委员和其他列席人员提出意见。

2018年工作回顾

过去一年，面对各种风险挑战和繁重的发展任务，市政府紧紧围绕“四城”建设目标，坚持稳中求进工作总基调，聚力追赶超越，推动高质量发展，较好完成了市十五届人大三次会议确定的各项目标任务。全市实现地区生产总值2265.16亿元，增长7.2%，三次产业结构比7.2∶63.3∶29.5；完成财政总收入231.82亿元，增长10.6%，地方财政收入87.76亿元，增长8.2%；城乡居民人均可支配收入分别达到31802元和11936元，增长8.2%和9.3%，经济社会发展迈出新步伐。

产业支撑更加有力，发展质量明显提高。工业强市建设加快推进，全市规模以上工业总产值增长8.5%。吉利发动机一期35万台生产线实现量产，全市整车产量达到20.75万辆，钛产业规模稳居全国首位、世界第二。烽火通信荣获中国工业大奖，中铁宝桥、法士特、汉德车桥获国家科技进步一等奖。国家级研发中心或分中心达到17个，陕西省智能机器人创新中心获批筹建。军民融合“宝鸡模式”被《人民日报》专题报道。111个工业技改项目顺利推进，新增高新技术和瞪羚企业24户。东岭集团成为全省首个总收入过千亿元的民营企业。现代农业加速发展，5个万亩特色农产品基地全面建成，16个现代农业园区被确定为全国农村创业创新园区。我市被授予“中华蜜蜂之乡”，“宝鸡苹果”成功注册国家地理标志证明商标。眉县获评全国十佳乡村振兴示范县，凤县入围全国农村一二三产融合发展先导区创建行列，太白高山蔬菜供港进澳，陇县食用菌出口韩国。旅游市场持续火热，全年游客接待量、旅游综合收入分别增长27.5%和32.6%。扶眉战役纪念馆改造工程顺利完工，周文化景区、大东湖景区、中国工合纪念馆等建成开放，太白山国际旅游度假区被命名为“中国温泉之乡”，九龙山创建为国家4A级旅游景区，陇县荣膺中国优秀生态旅游县，太白县、陈仓区入围“2018中国最美县域”，千阳县成为全国百佳乡村旅游目的地。商贸服务业发展势头良好，全市社会消费品零售总额稳居全省第二。陈仓老街、三国小镇等旅游商业街区建成开放，西秦刺绣、宝鸡面皮、凤翔泥塑、马勺脸谱等名优产品热销海内外，我市连续两年被评为“全国优秀电子商务城市”，麟游县、太白县跻身国家电子商务进农村示范县。

项目建设扎实推进，有效投资不断扩大。510个重点项目完成投资1045亿元，带动全社会固定资产投资增长12.1%。西凤酒城一期酒库扩容工程完工，麟游低热值煤发电厂并网发电，航天产业基地、中车时代高新生产基地加快建设，宝鸡综合保税区等项目前期工作取得积极进展，全省重点项目现场会在我市召开。全年实施招商引资项目860个，新开工10亿元以上大项目11个，实现招商引资到位资金1381亿元，增长15.8%。先后在京津冀、苏沪浙、长江中游、粤港澳、新疆等地区开展10余场次主题招商和旅游推介活动，招商引资的影响力和效果不断拓展提高。

三大攻坚战成效明显，营商环境日益优化。脱贫攻坚纵深推进，实施到村产业扶贫项目1124个，易地扶贫搬迁2108户，危房改造5241户，全市7.6万贫困人口实现脱贫，355个贫困村退出，5个贫困县摘帽工作完成市级核查，全国社会组织扶贫现场观摩会在我市召开，驻村帮扶、就业帮扶、扶贫资金绩效管理等工作经验全国交流，农村公共基础设施“1+7”物业式管理办法全省推广，荣获首批全国脱贫攻坚优秀城市。污染防治成效显现，市区空气质量优良天数、城市水环境质量，分别位居关中城市和渭河流域前列。秦岭生态环境保护专项整治取得阶段性成效，拆除违建11.8万平方米，恢复植绿35.3万平方米。金融服务实体经济水平不断提升，全年新增贷款157亿元，商业银行不良贷款率保持低位，政府债务风险总体可控。“放管服”改革持续深化，县区政务服务中心全部建成投用，市县两级政务服务平台实现互联贯通，办理时间整体压缩50%，不动产登记便民延时服务、“星期六对外办公”等创新举措在全省推广。

基础设施更趋完善，城市品质稳步提升。全国性综合交通枢纽建设成效明显，宝鸡机场前期工作取得实质进展，绛帐至汤峪、陇县至陕甘界、连霍高速市区过境线实现通车，阳平铁路物流基地一期即将建成投运，眉县荣获“四好农村路”全国示范县。城市品质提升“十大行动”加快实施，大庆路、姜谭路综合改造，以及植物园、联盟、阳平三座渭河大桥建设进展顺利，省人防综合训练基地建成竣工，完成城市“五拆”13.9万平方米，改造老旧小区231万平方米，惠及群众3.3万人，保障性住房考核位居全省前列。国家公交都市创建给市民带来更多方便。绿色宝鸡“九大工程”扎实推进，实现省级绿化模范县全覆盖，陈仓区通过全国绿化模范区创建验收，“三北”防护林体系建设受到国务院表彰，扶风七星河、岐山落星湾建成国家湿地公园。重点区域开发加力提速，宝鸡高新区在157个国家高新区排名中提升至第52位，蟠龙新区龙翔大道全线贯通，蔡家坡跻身全国综合实力千强镇。新型智慧城市建设步伐加快，12345市民服务热线正式开通，市区重点公共场所无线网络实现全覆盖。

改革开放步伐加快，发展活力显著增强。农村“三权分置”改革深入推进，县级产权交易市场在全省率先实现全覆盖，农村土地流转率达到30.7%，“1234”改革模式全省推广。国企改革稳步实施，完成国有商贸流通企业整合重组，“三供一业”、国有企事业单位公车改革基本完成。对外开放水平不断提高，电子口岸公共服务平台建成投用，国际贸易“单一窗口”货物申报实现全覆盖。“海外宝鸡”行动实现新突破，红旗民爆吉尔吉斯生产线正式投产，麦克传感器德国分公司投入运营，与全球156个国家和地区建立贸易往来，宝鸡的国际朋友圈不断扩大。

民生保障持续改善，群众得到更多实惠。全市财政民生支出达到270.1亿元，占总支出的81.8%，市政府承诺的十件实事全部完成。城镇新增就业5.5万人，农村劳动力转移就业98.65万人，社会保险标准化先行城市创建走在全国前列。城乡义务教育一体化改革深入推进，新建、改扩建中小学校68所，普惠性幼儿园占比提前两年达到国家标准。文化知名度不断提升，在麟游拍摄的电视剧《黄土高天》央视一套播出，8部作品获评冰心散文奖等全国性奖项，宝鸡珍贵文物在欧美展出。“健康宝鸡”加快建设，在全省率先完成城镇居民基本医保与新农合制度的整合，全国地方病专项防治工作推进会在我市召开。凤县新时代文明实践中心建设入选全国试点，33人荣获“中国好人”和“陕西好人”称号。金台区成为全国社区治理和服务创新实验区，眉县被评为全国农村幸福社区建设示范县，我市被确定为中央财政支持的居家和社会养老服务改革试点地区。节约集约利用土地工作受到国务院通报表彰。安全生产形势总体平稳，扫黑除恶专项斗争取得有效进展，扶风县、金台区荣膺全国法治县区创建先进单位，连续9年获评全省综治工作优秀市，公众安全感和社会满意率不断提升。

与此同时，审计、统计、物价、粮食、信访、供销、气象、档案、地志、市场监管、民族宗教、国家安全、防震减灾、妇女儿童、侨务外事、红十字会等工作也取得了新的成绩。

各位代表，过去一年，我们相继举办和承办了宝鸡发展大会、“一带一路”国家经贸合作洽谈会、中国汽车零部件行业年会、石油装备产业国际洽谈会、第七届中国西部跨国采购会、中国国际铅锌年会、国际机器人暨智能制造展览会、全国村长论坛、全国职工职业技能大赛等重大活动，更多宝鸡制造、宝鸡声音赢得赞誉、走向国际，城市知名度、影响力大幅提升；我们成功举办了全国太极拳公开赛、水球冠军赛、女子举重冠军赛、麟游和陈仓半程马拉松赛等体育赛事，全面展现了运动之城、活力宝鸡的独特魅力，“看中国，来宝鸡”品牌更加靓丽。

这些成绩的取得，是市委正确领导的结果，是市人大、市政协监督支持的结果，是全市上下干群同心、创新实干的结果。在此，我代表市政府，向人大代表、政协委员、各民主党派、各人民团体、社会各界人士，向驻宝企事业单位、人民解放军、武警驻宝部队全体官兵，表示崇高的敬意和诚挚的感谢！

在总结成绩的同时，我们也清醒的看到，全市经济社会发展中还存在一些困难和问题：产业结构调整步伐还需加快，发展质量和效益有待提升；三大攻坚战任务依然艰巨，营商环境需要下势优化；区域发展不够平衡，民生事业还有不少短板；一些政府公务人员的工作与新时代追赶超越的要求还不适应。对于这些问题，市政府将高度重视，强化措施，创新机制，切实加以解决。

2019年主要工作

今年是新中国成立70周年，也是决胜全面建成小康社会的关键之年。当前，我国经济面临下行压力，但经济长期向好的基本面没有改变，中央将继续实施积极的财政政策和稳健的货币政策，大力培育国内市场，持续推进创新驱动、乡村振兴、军民融合、西部大开发等战略，加大基础设施等领域补短板力度。国家出台的物流枢纽布局和建设规划，把宝鸡确定为生产服务型物流枢纽承载城市；省政府印发的《关中平原城市群发展规划》实施方案，将宝鸡定位为副中心城市。只要我们坚定信心，主动作为，以干克难，就一定能够抓住和用好各种机遇，应对和战胜各种风险挑战，实现全市经济社会平稳发展。

按照市委十二届五次全会部署，今年政府工作的总体思路是：以习近平新时代中国特色社会主义思想为指导，全面贯彻党的十九大、十九届二中三中全会和省市委全会精神，坚持稳中求进工作总基调，坚持新发展理念，坚持推动高质量发展，坚持以供给侧结构性改革为主线，坚持深化市场化改革、扩大高水平开放，紧扣追赶超越和“五个扎实”要求，全面落实省委“五新”战略任务，持续打好三大攻坚战，大力发展“三个经济”，进一步稳就业、稳金融、稳外贸、稳外资、稳投资、稳预期，增强人民群众获得感、幸福感、安全感，加快“四城”建设步伐，谱写新时代宝鸡追赶超越新篇章。

全市经济社会发展主要预期目标是：地方生产总值增长7.5%，全社会固定资产投资增长11%，规模以上工业增加值增长8%，社会消费品零售总额增长9%,地方财政收入增长5%以上，城乡居民人均可支配收入分别增长7.5%和8.5%。

一、持续做强工业，加快建设装备制造名城

认真落实李克强总理“装备中国，走向世界”的要求，深入实施“1553”行动计划，提升产业链水平，推动工业向价值链中高端迈进。

做大做强优势产业集群。推动汽车及零部件产业快速发展，围绕“一城两区五基地”建设目标，强化保障服务，重点抓好吉利产品升级、陕汽20万辆商用车生产基地、通家扩能技改等整车项目。加快法士特—伊顿轻型变速器、20万辆汽车零部件涂装生产线、汉德车桥宝鸡新基地、拓普汽车内饰等一批汽车零部件项目建设，努力形成配套宝鸡、服务陕西、辐射中西部、出口海外的汽车零部件产业集群。推动装备制造产业提升水平，抓住国家扩大管道运输的机遇，以宝石钢管为龙头，争取管材制造业快速增长，支持石油装备企业开拓国际市场。深度融入高铁“走出去”战略，推进中铁电气化宝鸡科技产业园、中车轨道交通产业园、凤县新能源空中列车西北制造基地、金台区中车时代扩能提升等项目建设。加快实施爱默生电梯、新能源叉车等重点项目，促进装备制造产业提速发展。推动钛及新材料产业扩大规模，加快推进宝钛等龙头骨干企业技术改造和产业升级，抓好高新区钛谷新材料循环再利用产业中心、核级锆材、钛及钛合金军民融合，以及眉县钛材精密铸造、智能装备生产线等项目建设，进一步巩固提升中国•钛谷影响力。推动烟酒食品产业提质增效，加快西凤酒城一期、柳林酒业技改等项目推进，建成西凤酒文化创意产业园。支持宝烟优化产品结构，抓好青啤15万吨纯生线技改扩产、蒙牛乳业扩能改造、陇县沙棘深加工等项目建设。推动能源化工产业迈向高端，推进麟游绿色循环经济工业园建设，力争总规模1040万吨的招贤、园子沟煤矿正式投产，做好坑口发电、中宝一矿、李家河煤矿、40万吨煤制乙二醇等项目前期工作。抓好徐矿西部生产基地建设，加快实施凤翔长青工业园90万吨甲醇、二甲醚及甲醇下游深加工项目，延伸煤化工产业链条。

培育壮大战略新兴产业。加强与中航、西飞等军工企业合作，抓好宝成高精度航姿系统产业化等项目建设，打造航空航天设备产业链。实施眉县6万吨、千阳3万吨锂电池石墨负极材料等项目，有序发展风电能源，加速新能源及节能环保产业化发展。支持渭滨区发展传感器产业，抓好华天电子科技半导体、烽火应急通信指挥系统、长岭迈腾电子芯片等项目建设，推动电子信息和光通信产业加速发展。提升扶风生物医药产业发展水平，推进华大基因测序医疗中心、西北机器厂智能放疗设备产业化等项目，促进生物医药与健康设备产业融合发展。抓好机器人智能制造、渭河工模具谐波减速器、秦川机床机器人关节减速器、宝鸡机床人工智能产品开发等项目建设，引导制造企业与生产性服务企业资源整合、业务融合，促进企业由设备提供商向集成服务商转变，推动宝鸡制造向“宝鸡智造”迈进。

改造提升特色产业。坚持绿色发展理念，推动建材、纺织服装、有色冶金三大特色产业转型发展。加快岐山建材、千阳环保新材料、常兴纺织产业结构调整，实施冀东水泥环保技改、凤县有色金属矿采选改扩建、眉县兴洲印染改造升级等项目，实现传统产业产品升级、提质增效。持续巩固“三去一降一补”成果，淘汰落后产能，积极引导企业实施兼并重组、债务重组和破产清算，稳妥处置“僵尸企业”，逐步实现市场出清。

催生发展新动能。大力推进国家创新型城市建设，聚焦“三个变革”，围绕骨干企业，发展产业集群，促进科技链、创新链、产业链、资本链和人才链融合发展。大力开展品牌建设，提高宝石机械、陕汽商用车、法士特齿轮等名牌产品知名度，不断增强市场竞争力。加强与清华、北大、西安交大、中汽研究中心等高校、机构合作，谋划建设汽车研究院、中航通飞宝鸡研究院等一批国家级创新平台。加快岐山国家级和陇县、麟游省级创新型试点县申报步伐，力争实现省级创新型试点县区全覆盖。加大“创客空间”、孵化基地和瞪羚企业培育力度，年内新增各类科技创新型企业20户以上。全力争创省级军民融合创新示范区，抓好马鞍山特种材料等58个军民融合项目建设，力争年内军民融合企业突破70户。全面实施总投资203.5亿元的85个工业技改项目，争取更多的新品开发、智能工厂、节能降耗项目进入中省技改专项资金支持序列，促进企业产品创新、技术创新和模式创新。实施人才强市战略，完善人才政策及体制机制，深化“智汇宝鸡•人才强企”“院士专家宝鸡行”“专家教授企业行”等活动，促进人才与资源、产业精准对接，多途径引进实用型、紧缺型及高层次人才。探索设立技能专家、首席技师、特级技师等岗位，通过技能竞赛等多种方式培养选拔技术工人，培育更多的新时代“宝鸡工匠”。

二、加速做大三产，持续推动经济结构优化

坚持把服务业作为补短板、调结构的重要环节，做火旅游、做旺商贸、扩大消费，加快建设历史文化名城。

构建文旅发展新格局。大力实施旅游“5+”战略，全力创建国家全域旅游示范市。支持太白山申报世界自然遗产，创建国家级旅游度假区和世界地质公园。抓好关山草原、“大水川-九龙山”一体化5A级景区和省级旅游度假区创建，加快推进鳌山滑雪场运动休闲度假区、长乐塬工业遗址公园、常青国际康养旅游度假基地、融创天朗文旅小镇等项目建设，打造一批精品旅游景区。加强旅游形象策划、品牌塑造和宣传营销，办好第八届文化旅游节。实施酒店业服务提升计划，加速构建交通互联、智慧旅游、标识系统等公共服务体系。积极推进周原、雍城遗址申遗工作，加快九成宫等考古遗址公园建设进度，启动张载文化产业示范区、法门寺博物馆改扩建等项目，创建国家文物保护利用示范区。推进体育产业规模化，抓好十四届全运会场馆改造提升，办好全国女子水球冠军赛、象棋公开赛等高水平赛事，打造活力之城、动感宝鸡。举办全国第十二届书法篆刻作品展，以及国际性石鼓文等文化品鉴、研究论坛，开展“名人讲宝鸡”系列活动，推动文化“走出去”，让文物“活”起来、文化“火”起来、旅游“热”起来。

做足消费升级大文章。高标准布局建设大型消费商圈，加快东岭等城市综合体建设进度，抓好南客站吾悦广场、高力欧陆购物中心等项目建设，提升胜利塬、太阳市等夜间经济示范点吸引力，打造一批高品位的步行街。实施消费升级行动计划，落实国家促进汽车、家电等产品消费措施，挖掘农村消费潜力。完善住房租赁、家政服务等配套政策，增加高品质产品和服务供给。加快培育特色商贸小镇，带动传统商贸转型升级。深化“最中国美食城市”建设，继续开展宝鸡名优特产展示展销活动。全年培育限上商贸流通企业40户。

推动新兴服务业快速发展。按照“互联网+制造+服务”的思路，着力打造工业设计、信息服务、商务会展等现代服务业新引擎，加快建设宝鸡金融文化商务区。围绕建设生产服务型国家物流枢纽，推进保税物流中心、宝鸡名品电商中心、西部跨国采购中心等平台建设，加快阳平铁路物流基地、西凤现代物流园、传化物流公路港等项目建设进度，积极引进国内外知名物流企业在宝鸡设立总部或者分支机构，不断壮大现代物流产业。大力创建国家电子商务示范市，抓好电子商务进农村综合示范县建设，支持陈仓物流电商产业园创建国家级电子商务示范基地，推进太白县农产品电子商务物流配送中心等项目建设，促进县域新零售业态发展。加快大数据产业发展，创建一批省级以上文化产业示范基地，推动文化创意、动漫游戏等新兴业态发展。制订优惠政策，积极发展总部经济，努力实现新增100亿元以上产值目标。

三、聚力脱贫攻坚，全面推进乡村振兴

以实施乡村振兴战略为引领，抓重点、补短板、强弱项，促进农业强、农民富、农村美。

大力发展现代农业。实施农业特色产业“3+X”工程，做好“三绿文章”，实施“三品工程”，推进“三产融合”，建设特色现代农业强市。持续壮大优势产业，深入开展农业结构调整大会战，突出标准化建园、板块化推进、集中连片成带发展，切实保障粮食安全，打造5个万亩产业示范基地和百万只奶山羊基地。扎实推进强县兴村示范工程，力争年内1-2个县区成为全省“现代农业强县”，建设一批农业产业强镇、产业富民强村。推广苹果“千阳模式”和优质猕猴桃生产技术，扩大优质果品和苗木面积。创建陇县全省奶山羊全产业链示范县，扶持千阳、陈仓、凤翔等奶山羊养殖、加工重点县区发展。持续抓好非洲猪瘟防控。年内发展优质订单小麦70万亩，标准化果园10万亩，设施蔬菜、高山蔬菜等4万亩，奶山羊20万只。促进质量品牌提升，扎实开展质量兴农提升行动，建立奶山羊、猕猴桃、矮砧苹果等产业规范，新认证无公害农产品产地、产品40个。积极参加全省“三年百市”活动，在徐州等城市建立宝鸡优质农产品专营店柜，办好全国猕猴桃产业发展大会、中国“槐花•蜜蜂”节、全国林麝产业发展论坛等活动，不断提高我市农产品的品牌知名度。积极推进三产融合，加快眉县国家现代农业产业园建设，支持陇县、凤翔、太白围绕区域优势产业，创建省级、国家级现代农业产业园区。抓好7个农产品加工园区提升，新建岐山农产品加工园区，支持扶风等农产品加工园区创建省级示范园区。做好“大棚房”专项清理整治巩固工作。

聚力推进脱贫攻坚。坚持精准扶贫、精准脱贫方略，高质量打赢脱贫攻坚战。狠抓脱贫退出，做好5个贫困县摘帽省级评估、国家抽查工作，完成43个贫困村、1.7万贫困人口稳定脱贫退出，实现全市整体脱贫目标。强化产业就业支撑，抓好104个“嵌入式”产业脱贫示范基地和100个扶贫龙头企业建设，巩固完善以“公益特岗为主、管护公岗为辅、公益专岗为补”的就业扶贫“宝鸡模式”，实现有劳动能力贫困人口产业就业扶贫全覆盖。巩固脱贫成果，继续做好脱贫户的精准帮扶和退出村基础设施建设，抓好移民搬迁点管理服务，全面提升农村公共基础设施“1+7”物业式管理水平。扎实做好各类特殊困难群体脱贫工作，实现“应保尽保、应兜尽兜”。研究解决收入水平略高于贫困户边缘群体的收入不稳定、缺乏政策支持等问题。深化苏陕扶贫协作，巩固社会扶贫成果。以“扶志六法”为抓手，着力激发贫困群众内生动力。

加快建设美丽乡村。全域推进美丽乡村创建，因村制宜，做亮特色，建设一批省级乡村振兴示范村，争创一批省级美丽宜居示范村。大力实施农村人居环境整治三年行动，持续推进“厕所革命”，做好农村生活污水处理，90%以上行政村生活垃圾得到有效治理，让农村更加干净整洁。抓好“四好农村路”省级示范县创建。持续推进农村城市化供水工程，改善30万人饮水条件。加快推进太白龙王河水库、石沟水库建设，力争普化水库、通关河水库、南峡沟水库开工。抓好“气化宝鸡”二期工程，加快“电化宝鸡”步伐，不断改善农村基础设施条件。实施好退耕还林、天然林保护和防护林体系建设工程，统筹推进“森林四围”建设，抓好乡村绿化美化，推进主干道路、出入境口及村庄绿化提升改造，实现乡村主干道路两侧植树种花全覆盖。

四、加快城镇建设，不断增强辐射带动能力

继续实施城市品质提升“十大行动”，进一步增强城市吸引力、承载力和竞争力。

提升城市服务功能。加快编制新一轮城市总体规划和城市总体设计，依法严格控制规划执行与项目设计，科学引领城市建设和发展。积极争取连霍高速市区段市政化改建，推动蟠高快速干线和陈仓、虢磻、钛谷、清溪、团结五座渭河大桥建设，上半年实现植物园、联盟、阳平及虢磻路跨陇海铁路大桥建成通车，争取城市轻轨国家立项，加快构建“东西快、南北畅”城市交通体系。高标准建设国家公交都市。深入开展城市“双修”，加大海绵城市建设力度，继续实施一批市区停车场、老旧小区改造、城市“五拆”、新能源汽车充电桩等民生项目，包装推进一批较大范围的整片区旧城改造项目。加强城市精细化管理，深入推进城管执法体制改革，加快城乡环卫一体化进程，促进城市环境共治共享。

推进重点区域开发。做强高新区，对标长三角和珠三角城市，全力推进科技新城、高铁新城，以及太公湖生态文化区等项目建设，加快推进汽车及零部件、钛及新材料等产业发展，努力建设西部创新之城和全国一流园区。建设新蟠龙，围绕打造教育新城、生态新城、康养新城，加快宝鸡大学、雅居乐国际双语学校、西安交大蟠龙医院、市委党校搬迁，以及绿地、恒大商业综合体等项目建设。做大蔡家坡，加快推进镇级市培育，进一步完善市政道路、集中供热、污水处理，以及学校、医院、高档社区等配套设施，抓好专业化、标准化厂房及入驻项目建设，全力做大做强以陕汽商用车、通家、法士特为龙头的汽车产业链，坚定不移推进千亿汽车新城建设。

完善县域城镇体系。积极推进凤翔撤县设区与眉县撤县设市，加快扶风全国文旅融合示范县建设，巩固提升岐山全省十强县位次，支持陇县打造世界乳都、千阳建设中国有机苹果生产示范县，抓好凤县、太白5A级县城建设，开展麟游县城整体搬迁前期工作。加快汤峪、法门、柳林3个全国特色小城镇、九成宫等4个省级文化旅游名街区，以及19个省级旅游特色名镇建设，加大对蔡家坡国家级、眉县省级新型城镇化试点支持力度，力争每个县区建成2-3个工业重镇、商贸强镇、休闲名镇。

加快对外通道建设。围绕建设全国性综合交通枢纽，抓好“一场三铁六条高速”建设，着力构建综合交通网。力促宝鸡机场上半年开工，做好西安至法门寺、法门寺至西安咸阳国际机场城际铁路建设环境保障工作，加快法门寺至宝鸡城际铁路、宝汉巴南铁路、宝中复线铁路前期工作。推进宝鸡至坪坎、太白至凤县、旬邑至凤翔高速公路项目，力争眉县至太白、麟游至绛帐、周至到凤翔高速公路宝鸡段上半年开工，积极推动北绕城高速公路规划建设。抓好麟游两亭至良舍等7条国省干线升等改造。

五、坚持生态优先，全力推进美丽宝鸡建设

积极顺应人民群众对良好生态环境的期待，着力解决环境突出问题，坚决打好青山、碧水、蓝天、净土保卫战。

全力守护青山净土。保持严管态势，以秦岭区域为重点，加快划定并严守生态保护红线。尽快修编秦岭生态保护总体规划和专项规划，抓好网格化、信息化监管平台建设，提高制度化、常态化监管水平。严格产业和项目准入，规范矿产资源开采利用、乡村旅游、农家乐等生产经营活动，按期完成采石企业治理，推动秦岭生态环境持续改善。大力开展土壤污染防治，做好凤县农用地土壤污染治理与修复技术应用试点，开工建设主城区垃圾焚烧发电项目。

强力保卫蓝天白云。准确把握汾渭平原国家主战场定位，以更高标准、更严措施、更大力度，改善大气环境质量。突出抓好工业污染治理，实施工业窑炉、挥发性有机物等重点行业污染治理升级改造项目，监督企业达标排放，继续推进高排放老旧机动车淘汰更新。加强“散乱污”企业治理，按照科学整治、分类施策、整体推进要求，“一企一策”做好“散乱污”企业治理工作，确保按期完成综合整治和转型发展任务。引导小微企业改善生产条件，升级工艺设备，进入园区高质量发展。扎实推进燃煤锅炉综合治理，对35蒸吨以下燃煤锅炉实现“清零”，同步做好清洁供热等替代工作；对35蒸吨及以上燃煤锅炉、城市建成区生物质锅炉，实施超低排放改造。切实强化扬尘治理，严格执行6个100%+红黄绿牌管理制度，科学有效落实各类建设工地控尘措施，不断扩大城镇街道机械化清扫作业面。深入开展煤炭削减，全面推行煤炭消费总量控制，在平原地区有序实施“电代煤、气代煤”，以及地热能、生物质能、太阳能等替代农村散煤取暖项目，抓好洁净煤供应工作，大幅度提升清洁取暖覆盖面。

倾力呵护碧水清流。全面落实河长制、湖长制，推进千河、清姜河、清水河等渭河、嘉陵江支流综合治理，强化水体污染补偿考核，促进河流水质稳定达标。加快高新科技新城、蟠龙新区、五丈原等污水处理厂建设，确保按期建成投运。大力推进市区、县城、工业园区和重点镇截污管网建设，努力做到污水全收集、处理全覆盖。扎实推进县级集中式饮用水源地专项整治，确保年底前全部整改到位。加快标准化砂厂建设进度，保障砂料充足供应，杜绝非法采砂行为。

六、深化改革开放，努力打造“一带一路”上的国际化城市

主动融入国家“一带一路”战略，进一步扩大对外开放，推动改革走深走实，不断激发新活力，再造新优势。

全面深化改革。扎实推进国企国资改革，建立和运营好国有资本投资、运营公司，积极推广宝钛“1+N”改革模式，稳步推进国有企业混合所有制改革。加快企业股份制改造步伐，提升国有资本证券化率。持续加强市属企业资产负债约束，着力化解企业债务风险，逐步解决国企改革遗留问题。增强微观主体活力，最大限度减少政府对资源的直接配置，破除各类要素流动壁垒，增强企业内在活力。全面完成行政机构改革任务，深化行政执法体制改革，统筹推进事业单位改革。落实“三权分置”改革配套政策，鼓励支持土地经营权有序流转，因地制宜实施农村“三变”改革，抓好陇县、岐山县全国农村集体产权制度改革试点。深化财税体制改革，突出地方主体税源培育，集中财力办大事，做到“三公经费”只减不增。

扩大对外开放。围绕发展“三个经济”，以会展中心、综合保税区、阳平铁路物流基地、现代临空经济区为支撑，在城市东区规划建设以现代物流、信息金融、商贸会展、保税仓储加工等服务为一体的宝鸡港务区。出台全面深化改革扩大开放的意见，加快“海外宝鸡”建设，实施宝光股份、宝鸡机床境外项目，争取宝鸡钢管阿拉木图生产线投产。深度融入“一带一路”，组织我市名优产品在广西凭祥口岸、莫斯科设馆展销。做好中欧国际货运宝鸡专列的服务保障工作，着力打造宝鸡自己的陆运口岸。积极承办国际国内重大会议和商务活动，办好中国西部跨采会、中国国际机器人暨智能制造展览会、中国汽车知识产权年会、中国钛谷•国际钛产业博览会和全国激光产业论坛，谋划好各类大型专业化会展活动，筹建宝鸡市工业品展览馆，深化与14个国内外友好城市合作交流，持续提升对外知名度和影响力。

壮大民营经济。紧紧围绕习近平总书记提出的六个方面政策举措，落实省委、省政府《推动民营经济高质量发展的若干意见》，制定支持民营经济发展的政策措施，积极构建清亲新型政商关系。进一步聚焦企业关切，破解企业投资生产经营中的“堵点”和“痛点”，全面落实减税降费政策，保障民营企业家合法权益，不断增强民营企业投资和发展信心。提升县域工业园区配套服务能力，继续加大标准化厂房建设力度，降低民营企业经营成本，以民营经济发展助推县域经济追赶超越。

创新金融服务。落实金融机构服务实体经济的一系列政策措施，扩大普惠金融覆盖面，抓好100户后备企业辅导服务，鼓励高新技术领域优质企业加快上市步伐，引导中小企业在“新三板”及陕西股权交易中心挂牌，支持优势企业利用发行债券等方式直接融资，提升多层次资本市场融资能力。全年实现新增贷款130亿元以上，社会融资规模达到2200亿元以上。分步设立10亿元民营企业纾困基金，把企业政策性融资担保基金逐步扩大到10亿元，着力解决民营企业融资难、融资贵问题。创新投融资方式，用足用活国家政策，盘活政府存量资源。加快政府融资平台市场化转型，严控政府隐性债务，打击非法集资、金融诈骗、恶意逃废债等行为，维护金融秩序稳定。

七、着力改善民生，不断提高群众幸福指数

坚持以人民为中心的发展思想，多谋民生之利，多解民生之忧，努力建设宜居宜业的美丽幸福城市，不断满足人民群众日益增长的美好生活需要。

完善社会保障体系。实现技能培训全覆盖，支持返乡下乡人员创业创新，千方百计促进创业就业，全年城镇新增就业4.3万人，城镇登记失业率控制在4.3%以内。提高城乡居民大病保险筹资标准，深化社会保险制度改革，实施“援企稳岗”护航行动，全面提升政策“兜底”水平。完善刚性支出救助机制，进一步加强低保、救助、慈善、残疾人，以及农村留守儿童、妇女、老年人关爱服务工作。实施全国居家和社区养老服务改革试点，抓好18个社区老年人日间照料中心和24个社区养老服务中心建设，启动一批老年公寓项目。完善住房保障体系，建成棚户区安置房5500套。

办好人民满意教育。启动义务教育优质均衡县创建，修编中小学布局调整规划，扩大教育开放，积极引进双语等高端教育资源，加强教育国际合作。逐步消除义务教育学校大班额，着力解决“乡村弱、城镇挤”、学生课业负担过重等问题，提高普惠性幼儿园占比。推进民办教育分类管理，支持和规范民办教育发展。加快市职业教育实训中心建设，整合教育培训资源，做强职业教育优势专业。实施新建、改扩建教学及运动场地等项目120个，继续抓好学校运动场地向市民开放工作。加强教师队伍建设，实现义务教育教师平均工资不低于公务员平均工资水平。

繁荣发展文化事业。积极做好省九艺节筹备工作，建成市文化艺术中心，启动宝鸡大剧院建设。加强县域图书馆、文化馆和农村、社区综合文化服务中心建设，打造一批文学、书法、摄影创作基地，提升公共文化设施服务能力。巩固全国文明城市创建成果，深化群众性精神文明创建，汲取周秦优秀传统文化和红色文化的丰厚营养，培育良好家风乡风行风。积极开展“文化进万家”活动，全年完成公益性演出1000场次。完善文化体制改革，有效统筹社会文化资源，支持文艺创作，切实加强市县文艺团队建设。

建设普惠健康宝鸡。深入推进健康学校、健康社区、健康家庭等八类健康细胞示范建设，全面提升城乡居民健康水平。抓好市中医医院扩建，完成市精神卫生中心建设。深化公立医院改革，实施公立医院服务能力提升工程，做实紧密型医联体和县域医共体，加快标准化村卫生室建设，把更多救命救急的好药纳入医保，着力解决群众看病难、看病贵问题。对标国际、国内，引进高精尖医疗团队，提高我市整体医疗水平。加强出生缺陷综合防治，做好地方病和重点传染病防治防控。实现国家卫生城市复审达标。

提升社会治理能力。重视“平安宝鸡”建设，持续深化扫黑除恶专项斗争，全面推进社区警务“四单一图”工作新模式，实施“雪亮工程”，不断提升群众安全感。加强制度建设和社会信用管理，提高社会治理法治化、智能化、专业化水平。抓好第七次城市社区换届选举工作，实施25个农村社区服务中心、8个省级标准化示范社区建设项目。深入开展“七五”普法，推动全社会尊法学法守法用法。加强退役军人服务管理，依法维护军人军属合法权益。细化社会稳定风险评估和矛盾纠纷排查化解机制，把信访突出问题和群众合理合法诉求处理好、解决好。实施从田间到餐桌、生产到消费全过程监督，开展农村假冒伪劣食品专项整治，切实保障人民群众饮食用药安全。强化应急保障能力建设，不断筑牢安全生产防线，坚决遏制重特大事故发生。

建设人民满意政府

新时代要有新气象，新年度要有新作为。我们将努力践行“加压奋进、追赶超越、创新实干”的工作理念，一心一意搞建设，聚精会神谋发展，努力创造无愧于时代、无愧于人民的新业绩。

聚焦项目谋发展。坚持以项目支撑发展规划，用项目落实思路目标，靠项目增强发展后劲。全市各级政府要把主要精力用在抓项目上，确保540个市级重点项目顺利实施，完成年度投资1000亿元以上。继续落实“五个一”工作机制，坚持重点项目工作月排名、季通报和专项述职制度，集中精力抓开工、抓进度、抓投产。抢抓国家实施积极财政政策和较大幅度增加地方政府专项债券规模的机遇，在乡村振兴、民生、交通、能源、水利、环保等基础设施短板领域，谋划上报一批重大投资项目，全年争取中省资金180亿元以上。放开投资准入门槛，推介一批符合国家产业政策、前期基础实、投资回报好的项目，鼓励民间资本和社会资本以PPP等方式参与项目建设。

创新方式抓招商。深入开展“产业招商年”活动，围绕装备制造、汽车及零部件、军民融合等优势产业集群强链补链，瞄准行业龙头和领军企业，精心谋划，全力推介，吸引产业、项目、人才、技术向宝鸡聚集。在继续做好区域性招商活动的同时，积极组织10个产业招商分局和4个驻外招商分局，开展专业化、精准化小分队招商。各县区要围绕产业特色，重点开展专题定向定点招商活动，切实提高招商成功率。年内，各县区、各开发区要引进投资10亿元以上产业项目2—5个，完不成任务的年终目标责任考核实行一票否决。

优化服务强保障。深入实施优化提升营商环境“10+3”行动，进一步加大“放管服”改革力度，深化“证照分离”改革，充分发挥市县两级行政审批局的作用，持续优流程、减材料、压时间、降成本，打造市场化、法治化、便利化营商环境。积极推进智慧城市建设，加快云计算、物联网、大数据、人工智能在行业管理服务中的推广应用。完善市县镇三级政务服务网，市、县政务服务大厅全部接入信息平台和网站，政务服务事项网上可办率不低于70%，100个高频事项实现“不见面审批”或“最多跑一次”。对各县区、市级各部门的企业和市民投诉，定期排名通报，开展“零投诉”机关创建活动。不断推进工程建设项目审批制度改革。通过等量置换、减量置换等方式，腾出环境容量指标。抓好农村土地复垦和城乡建设用地增减挂钩工作，盘活批而未供的存量土地资源，加快土地等要素市场化步伐，推动土地向大项目、好项目倾斜。

转变作风促落实。加强政治建设，树牢“四个意识”，践行“两个维护”，深入开展“讲政治、敢担当、改作风”专题教育。各级政府公务人员要加强理论武装和专业知识学习，优化知识结构，应对本领恐慌。持续深化“大调研、送服务、解难题”活动，建立重点项目、重点工作专项工作组推进机制，用新理念、新办法谋划推动工作、破解瓶颈制约。坚决防止和克服官僚主义、形式主义，杜绝有令不行、有禁不止现象。强化责任担当，建设一流机关，争创一流业绩，为投资创业者和基层群众提供方便快捷、优质高效的服务。进一步加强目标责任考核，要考出压力、考出动力、考出生产力。

不忘初心守清廉。各级政府公务人员要以人民至上的公仆情怀，时时处处为民着想，用自己的“苦和累”换来群众的“笑与乐”。自觉接受人大、政协和纪委监委监督，主动接受群众监督和社会监督。加快建设法治政府，推进政府职能法定化和事权规范化。认真落实党风廉政建设主体责任和“一岗双责”，健全廉政风险防控制度，加强对重点领域、关键环节、重要岗位权力运行的监督制约，进一步规范政府采购、土地出让、矿产开发、工程招投标等工作，严管公共资源交易，加大财政监管和审计监督力度，推动政府系统全面从严治党向纵深发展，努力打造为民、务实、清廉的公务员队伍。

各位代表，责任在心重千钧，使命在肩催奋进。让我们在市委的坚强领导下，以习近平新时代中国特色社会主义思想为指导，凝聚万众之心、汇聚各方之力，坚定信心，锐意进取，扎实工作，全力加快“四城”建设，以优异成绩迎接新中国成立70周年！
